# Red Blood Cell Abnormalities as the Mirror of SARS-CoV-2 Disease Severity: A Pilot Study

**DOI:** 10.3389/fphys.2021.825055

**Published:** 2022-01-20

**Authors:** Anthi Bouchla, Anastasios G. Kriebardis, Hara T. Georgatzakou, Sotirios P. Fortis, Thomas P. Thomopoulos, Leoni Lekkakou, Konstantinos Markakis, Dimitrios Gkotzias, Aikaterini Panagiotou, Effie G. Papageorgiou, Abraham Pouliakis, Konstantinos E. Stamoulis, Sotirios G. Papageorgiou, Vasiliki Pappa, Serena Valsami

**Affiliations:** ^1^Second Department of Internal Medicine, Attikon Hospital, Research Institute, National and Kapodistrian University of Athens, Athens, Greece; ^2^Laboratory of Reliability and Quality Control in Hematology (HemQcR), Department of Biomedical Sciences, School of Health, and Caring Sciences, University of West Attica (UniWA), Egaleo, Greece; ^3^Second Department of Pathology, Attikon University Hospital, National and Kapodistrian University of Athens, Athens, Greece; ^4^National Blood Center, Athens, Greece; ^5^Hematology Laboratory – Blood Bank, Aretaieion Hospital, National and Kapodistrian University of Athens, Athens, Greece

**Keywords:** COVID-19, red blood cell, disease severity, hemolysis, SARS-CoV-2, chest infiltration, free hemoglobin

## Abstract

**Purpose:**

Unraveling the pathophysiology of COVID-19 disease is of crucial importance for designing treatment. The purpose of this study is to investigate the effects of the disease on erythrocytes (RBCs) and to correlate the findings with disease severity.

**Materials and Methods:**

Hospitalized patients (*n* = 36) with COVID-19 and control group of healthy volunteers (*n* = 18) were included in the study. Demographic data, clinical, laboratory and chest Computed Tomography (CT) findings at time of admission were recorded. Laboratory measurements included: Hemoglobin (H b), indirect billirubin, LDH, D-Dimers, and plasma free hemoglobin (plasma free-Hb). On RBCs were performed: osmotic fragility (MCF), Free-Hb after mechanical stress (Free-Hb-MECH), intracellular RBC concentration of calcium ions (iCa^2+^), intracellular ROS (iROS), G6PD, intracellular active caspase-3 (RBC-caspase-3), IgG immunoglobulins (RBC-IgGs), which are bound on RBCs’ senescent neo-antigen proteins and RBC surface phosphatidylserine (RBC-PS).

**Results:**

The percentage of males was 50 and 66% and the mean age was 65.16 ± 14.24 and 66.33 ± 13.48 years among patients and controls respectively (mean ± SD, *p* = 0.78). Upon admission patients’ PO_2_/FiO_2_ ratio was 305.92 ± 76.75 and distribution of infiltration extend on chest CT was: 0–25% (*N* = 19), 25–50%: (*N* = 7), and 50–75% (*N* = 9). Elevated hemolysis markers (LDH and plasma free-Hb) were observed in patients compared to the control group. Patients’ RBCs were more sensitive to mechanical stress, and exhibited significantly elevated apoptotic markers (iCa^2+^, RBC-PS). Plasma free Hb levels correlated with the extend of pulmonary infiltrates on chest CT in COVID-19 patients. Surprisingly, patients’ RBC-iROS were decreased, a finding possibly related with the increased G6PDH levels in this group, suggesting a possible compensatory mechanism against the virus. This compensatory mechanism seemed to be attenuated as pulmonary infiltrates on chest CT deteriorated. Furthermore, RBC-IgGs correlated with the severity of pulmonary CT imaging features as well as the abnormality of lung function, which are both associated with increased disease severity. Lastly, patients’ D-Dimers correlated with RBC surface phosphatidylserine, implying a possible contribution of the red blood cells in the thrombotic diathesis associated with the SARS-CoV-2 disease.

**Conclusion:**

This pilot study suggests that SARS-CoV-2 infection has an effect on red blood cells and there seems to be an association between RBC markers and disease severity in these patients.

## Graphical Abstract

SARS-CoV-2 infection has significant effects on red blood cells that seems to be associated with disease severity in these patients. **(A)** Non-Intensive care unit hospitalized patients due to severe COVID-19 disease were included in this study (*n* = 36). **(B)** The control group included healthy subjects without renal or hepatic impairment, no diagnosis of anemia or myeloid malignancy, and no blood transfusion over the past 3 months (*n* = 18). **(C)** Demographic data, clinical, laboratory and chest Computed Tomography (CT) findings at time of admission were recorded. Hematological tests, standard biochemical analysis of serum components were performed both in the patient and in the control group and coagulation analysis were evaluated in the patient group. A peripheral blood smear examination was also performed in all patients and control samples. **(D)** Plasma free hemoglobin and red blood cell (RBC) osmotic fragility and free hemoglobin (Free-Hb) after mechanical stress (MECH) by mechanical hemolysis were assesed in both patients and controls. Intracellular (i) calcium (iCa2 +) levels, RBCs phosphatidylserine exposure (PS), RBC membrane-bound immunoglobulins G (IgGs), and activate RBC caspase-3 levels as cell clearance and apoptotic markers were measured by flow cytometry. A spectrophotometric assay was used to quantify the activity of G6PD enzyme. Intracellular RBC reactive oxidative species (iROS) were assesed in both patients and controls. **(E)** Elevated hemolysis markers (Free-Hb) and Lactate Dehydrogenase (LDH) were observed in patients compared to the control group. Patients’ RBCs were more vulnerable to mechanical stress, and exhibited significantly elevated apoptotic markers (iCa2 + and RBC-PS+). Erythrocyte iROS levels were significantly lower in the patients’ group compared to healthy controls. This finding possibly correlates with the increased G6PDH activity levels in this group, suggesting a compensatory mechanism against the virus. **(F)** Patients’ D-Dimers correlated with RBC-PS +, implying a possible contribution of the erythrocytes in the thrombotic diathesis associated with the SARS-CoV-2 disease. **(G)** Free-Hb levels correlated with the extend of pulmonary infiltrates on chest CT in COVID-19 patients. RBC-IgGs+ correlated with the severity of pulmonary CT imaging features as well as the abnormality of lung function, expressed by the PO2/FiO 2 ratio. This finding suggests that hypoxia may lead to RBC senescence.

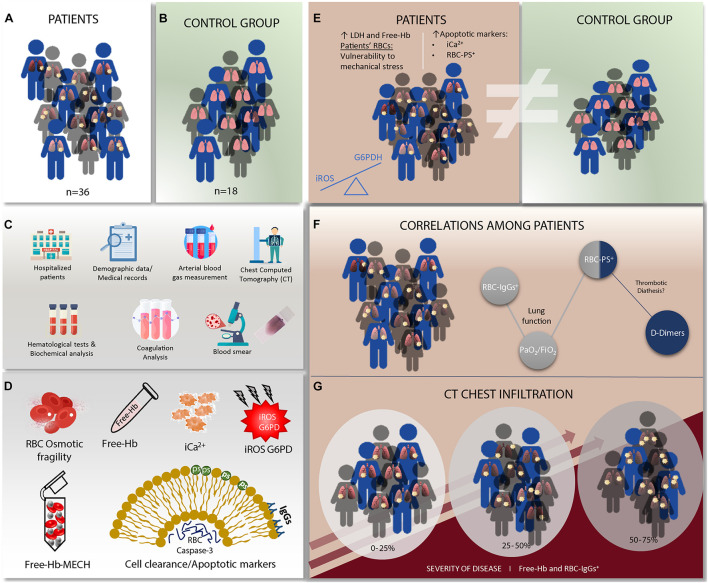

## Introduction

SARS-CoV-2 pandemic has demonstrated a devastating effect on humanity since its outbreak in 2019. Rigorous efforts have been made to describe its pathophysiology, in order to design more effective treatments. The virus’s impact on various systems besides the lungs has been acknowledged, involving the heart, gastrointestinal tract, nervous system, kidney, coagulation, and endothelium (Iba et al., 2020; Puelles et al., 2020; Su et al., 2020). A variety of hematological parameters, including lymphopenia and elevated D-Dimers can be observed in patients with COVID-19 infection (Rahi et al., 2021). Anemia has been reported as an independent predictive factor of disease severity in COVID disease (Bellmann-Weiler et al., 2020), with a number of studies consistently showing that increased Reb blood cell Distribution Width (RDW) predicts worse outcome in COVID-19 patients (Foy et al., 2020; Banon et al., 2021; Soni and Gopalakrishnan, 2021).

Red blood cells (RBCs) are the transporters of O_2_ throughout the body and they are known to be lysed in the case of infections, especially when complicated with sepsis (Bateman et al., 2017). Previous research has shown that the virus can invade the RBC via interaction of its S1 spike protein with RBC CD147 (Wang et al., 2020) and the band-3 protein (Cosic et al., 2020). Viral spike protein and complement activation products have been detected on the RBC cell surface of COVID-19 patients, and have been thought to affect RBC rheology, resulting in intravascular thrombosis and associated lung injury (Lam et al., 2021).

Moreover, it has been hypothesized that in COVID-19 infection, hypercoagulability and the inflammatory state may affect RBCs, causing their membrane to become more fragile and less elastic (Grobler et al., 2020). Finally, it has been shown that increased serum ferritin can cause hyperactivated interactions between RBCs and platelets in COVID-19 patients, suggesting a link between RBC alterations and thrombosis in COVID-19 disease (Venter et al., 2020).

The purpose of the present study is the investigation of the effects of SARS-CoV-2 on red blood cells’ physiology as well as their association with disease severity. Additionally, this study aims to elucidate the possible mechanisms behind this association.

## Materials and Methods

### Subjects

Non-Intensive care unit (ICU) hospitalized patients due to severe COVID-19 disease were included in this study from March 2021 to May 2021. The control group included non-COVID-19 patients without renal or hepatic impairment, no diagnosis of anemia or myeloid malignancy and no blood transfusion over the past 3 months. EDTA and serum samples were obtained from all participants as per study protocol (COVID-19 patients and controls). Correlations of the laboratory results with disease severity assessed with chest CT imaging and the PaO_2_/FiO_2_ ratio were performed in COVID-19 patients. CT imaging and PaO2/FiO2 ratio were performed upon patient admission. All the rest laboratory measurements were performed on the same day at various time points during patient hospitalization. The study was conducted according to the guidelines of the Declaration of Helsinki, and approved by the Institutional Review Board (or Ethics Committee) of National and Kapodistrian University of Athens (decision number: 145/8-3-2021 on 08-04-2021).

### Hematological, Biochemical, and Coagulation Analysis

Hematological tests [including WBC (white blood cell) count, Neutrophils, Lymphocytes, Monocytes, Eosinophils, Basophils, RBC (red blood cell) count, Hemoglobin (H b), Hematocrit (Hct), MCV (mean cell volume), MCH (mean corpuscular hemoglobin), MCHC (mean corpuscular hemoglobin concentration), RDW (RBC distribution width), PLT (platelet count), and MPV (mean platelet volume)] and standard biochemical analysis of serum components [glucose, urea, creatinine, uric acid, calcium, phosphorus, potassium, sodium, chlorine, magnesium, iron, ferritin, proteins, albumin, serum glutamyl oxalate transaminase, serum glutamyl pyruvate transaminase, gamma-glutamyl transferase, alkaline phosphatase, total bilirubin, indirect bilirubin, direct bilirubin, amylase, lactate dehydrogenase (LDH), high sensitive C-reactive protein (CRP), and interleukin-6 (IL-6)] were performed both in the patient and in the control group and plasma levels of cross-linked fibrin degradation products (D-dimers) and fibrinogen were evaluated in the patient group. A peripheral blood smear examination was also performed in all patients and healthy control samples. A sample of 10 μL EDTA blood was used for the preparation of blood smears, followed by May-Grünwald-Giemsa staining. More specifically, after the preparation of the blood smear, it was left to air dry and then it was immersed in pure methanol for fixation, for 5 min. The slide was then soaked into May-Grünwald stain solution for 4 min followed by Giemsa staining for 15 min. The excess of staining was removed by rinsing with water.

### Lung Function Examination

In the patient group, lung function was determined through the measurement of PO_2_/FiO_2_ ratio and the percentage of infiltration of chest computed tomography (CT) (in quartiles: 1–25, 25–50, 50–75, and 75–100%). Quantification of the extend of lung infiltrates was performed using the Covid Visual Assessment Scale (Arkoudis et al., 2021).

### Measurement of Intracellular ROS, Ca^2+^, and Activated Caspase-3 Levels

Intracellular ROS (iROS) and calcium (iCa^2+^) levels were measured by flow cytometry as previously described (Pavlou et al., 2021). For the detection of cleaved caspase-3 (RBC caspase-3), red blood cells were washed three times with phosphate-buffered saline solution (PBS) and were resuspended in PBS at a concentration of 4% Hct. For cell fixation, cells were incubated in 0.05% glutaraldehyde solution for 10 min in room temperature. RBC suspension was then incubated in PBS/Tween 0.5% solution along with PE Rabbit anti-active caspase-3 antibody, for 20 min in room temperature. Reaction was stopped by the addition of PBS solution. Antibodies used for flow cytometry were obtained from BD Biosciences (San Jose, CA, United States). Percentage of activated caspase-3 positive cells were determined by flow cytometry in a FACSCanto II Cytometer (BD Biosciences, San Jose, CA, United States). Data analysis was performed using the BD FACSDiva™ Software. A minimum of 30,000 events was acquired for RBCs on each sample.

### Detection of Red Blood Cell Membrane-Bound Immunoglobulins G and Phosphatidylserine Exposure

RBCs phosphatidylserine exposure (PS^+^ RBCs) was performed by using phycoerythrin (PE)-Annexin V apoptosis kit and RBC-gating marker, fluorescein isothiocyanate (FITC)-conjugated anti-CD235 (BD Biosciences, San Jose, CA, United States) and was determined by flow cytometry in a FACSCanto II Cytometer as previously described (Tzounakas et al., 2021).

For the detection of senescent neoantigen in red blood cells’ surface, the percentages of red blood cells that were positive on membrane-bound (Immunoglobulins G) IgGs (IgG^+^ RBCs) were measured. RBCs resuspended in PBS/0.1% BSA solution at a concentration of 0.1% Hct, were incubated with fluorescein isothiocyanate (FITC)-conjugated anti-IgG (BD Biosciences, San Jose, CA, United States) along with the RBC-gating marker, allophycocyanine (APC)-conjugated anti-CD235, for 40 min in room temperature. Reaction was stopped by the addition of PBS solution. A minimum of 30,000 events was acquired for RBCs on each sample.

### Hemolysis Tests and G6PD Activity

Plasma free hemoglobin (plasma free-Hb) was calculated by the method of Harboe (1959). More specifically, after a centrifugation of total blood at 1500*g*, plasma supernatant was doubly centrifuged at 1000*g* for 10 min. Supernatants were diluted in distilled water and incubated at room temperature for 30 min. Absorbance was measured at 380, 415, and 450 nm. The formula 2*OD415 - (D380 + D450)* dilution * 1000/1.655*8.60 = was used for the calculation of mg Hb/100 ml. Osmotic fragility test was performed as previously described (Tzounakas et al., 2017).

Mechanical hemolysis being referred to percent hemolysis of RBCs after exposure to mechanical stress (Free-Hb-MECH), was calculated as follows:

[((100 - Hematocrit) × (PFHb_control_))/Hb_aliquot_] × (dilution factor required to achieve 20% Hct)

where, PFHb_*control*_ is the mean plasma free hemoglobin concentration in the supernatants of the control (unrocked) samples, and Hb_*aliquot*_ is the mean hemoglobin concentration of the RBC aliquots at a hematocrit of 20% (Harm et al., 2012).

A spectrophotometric assay was used to quantify the activity of G6PD enzyme (Ben Srl Biochemical Enterprise, Milan, Italy).

### Statistical Analysis

A chi-square test was performed in order to check for imbalances between patient and control group regarding participants’ gender. Assuming non-normality of the parameters at study, Kruskal Wallis tests for two groups were performed for the evaluation of differences in parameters across subject groups. For the detection of differences in parameters across different classes of chest CT infiltrations in the patient group, Kruskal Wallis tests with Dunn’s test for multiple comparisons were performed. Finally, Spearman’s Rho was calculated in order to find an association between RBC parameters and PaO_2_/FiO_2_ ratio in the patient group. All values are expressed as median (25–75% interquartile range-IQR), unless stated otherwise. Significance was accepted at p values less than 0.05. Statistical analysis was performed using STATA MP-64 16.0.

## Results

In this study, hospitalized SARS-CoV-2 patients (*N* = 36) as well as an age and gender-matched healthy control group (*N* = 18) were included. Regarding their demographic characteristics, the group of patients consisted of 50% males, whereas in the control group the percentage of males was 66%, (*p* = 0.245). The mean age was 66.33 ± 13.48 years in patients and 65.16 ± 14.24 in healthy subjects (mean ± SD, *p* = 0.78). Drugs administered for hospitalized SARS-CoV-2 patients included remdesivir, dexamethasone, low-molecular weight heparin and broad-spectrum antibiotics, based on physician decision. There were no reports of drug- induced hemolysis in patient’s records. Neither patients nor controls received concomitant medications that could impact free hemoglobin, according to their medical records.

The main hematological profile and associated serum biochemichal and coagulation analysis of patients upon admission to the hospital and healthy subjects as well as patient’s respiratory assessments are presented in Table 1. Red blood cell characteristics in SARS-CoV-2 patients and in healthy subjects are presented in Table 2. SARS-CoV-2 patients showed lymphopenia, along with increased inflammation markers including ferritin, CRP and IL-6 levels. Also, increased LDH levels were observed in the patients in contrast to healthy subjects, who showed normal LDH values. No association between serum LDH, lymphocyte number, ferritin and D-dimers levels and disease severity was observed, however there was an association between fibrinogen levels and high sensitivity CRP (hs-CRP) in the patients’ group (r = 0.662, *p* = 0.003). No morphological red blood cell abnormalities were observed in either the healthy controls or the SARS-CoV-2 peripheral blood samples by light microscopy (data not shown).

**TABLE 1:** Hematological and serum biochemical characteristics and red blood cell hemolysis markers of patients with SARS-CoV-2 upon admission to the hospital and healthy subjects.

	Patients (*N* = 36)	Healthy subjects (*N* = 18)		
	MEDIAN (25–75% IQR)	MEDIAN (25–75% IQR)	NR	*P* value
**Hematological analysis**				
WBC (×10^3^/μL)	6.82 (5.24–8.95)	5.80 (4.48–6.49)	4.00–11.00	0.064
Neutrophils (×10^3^/μL)	5.34 (3.28–7.12)*	3.5 (3.11–4.46)	2.0–8.0	0.011
Lymphocytes (×10^3^/μL)	**0.88 (0.71**–1.42)*	1.4 (0.98–1.72)	1.1–4.0	0.015
Monocytes (×10^3^/μL)	0.56 (0.37–0.73)	0.35 (0.27–0.53)	0.1–0.9	0.064
Eosinophils (×10^3^/μL)	0.00 (0.00–0.03)**	0.15 (0.03–0.23)	0–0.8	0.000
Basophils (×10^3^/μL)	0.00 (0.00–0.01)**	0.05 (0.02–0.06)	0–0.2	0.000
RBC (×10^6^/μL)	4.67 (4.44–5.14)	4.66 (4.27–5.07)	4.2–6.1	0.873
Hb (gr/dL)	13.4 (12.10–14.40)	13.30 (12.30–14.80)	12.0–18.0	0.756
Hct (%)	40.4 (36.70–44.00)	42.20 (38.30–46.20)	37.0–52.0	0.396
MCV (fL)	85.2 (82.80–89.70)	89.70 (84.70–95.50)	80.0–99.0	0.297
MCH (pg)	28.7 (27.70–29.60)	29.10 (27.20–30.90)	27.0–31.0	0.808
MCHC (gr/dL)	33.3 (32.70–34.10)	32.00 (31.60–32.60)	33.0–37.0	0.832
RDW (%)	13.1 (12.40–13.90)	14.00 (13.35–17.23)	11.5–14.5	0.061
PLT (×10^3^/μL)	194.0 (164.0–294.0)	213.0 (137.0–279.0)	130.0–400.0	0.612
MPV (fL)	9.1 (7.90–10.40)*	8.00 (7.30–9.30)	7.2–11.1	0.033
**Serum biochemical analysis**				
Iron (mg/dL)	71.50 (44.0–87.5)	75.00 (59.00–110.00)	33–193	0.169
Ferritin (ng/mL)	**468.5 (292.0**–758.3)**	99.0 (41.0–515.0)	30–400	0.004
LDH (IU/L)	**331.0 (259.0**–396.0)**	188.0 (166.0–267.0)	135.0–225.0	0.000
Total Bil (mg/dL)	0.44 (0.43–0.64)	0.35 (0.29–0.51)	0.20–1.20	0.56
Indirect Bil (mg/dL)	0.17 (0.11–0.25)	0.13 (0.10–0.17)	<0.20	0.117
hs CRP (mg/L)	**76.70 (37.20**–165.00)**	2.05 (1.05–3.53)	0.0–6.0	0.000
IL-6 (pg/mL)	**44.70 (17.50**–109.00)**	7.90 (3.05–10.57)	0–17.4	0.000
**Coagulation analysis**				
Fibrinogen	592.8 (486.7–650.7)			
D-Dimers	649 ng/mL (441–1391.25)			
**Respiratory assessments**				
PO_2_/FiO_2_ ratio	305.92 ± 76.75 (mean ± SD)			
O_2_ per nasal canula	*N* = 22			
O_2_ per Venturi mask	*N* = 14			
CT 0–25%	*N* = 19			
chest 25–50%	*N* = 7			
infiltations 50–75%	*N* = 9			
**Erythrocyte mean cell fragility and plasma free hemoglobin**				
MCF (%)	0.43 (0.40–0.44)	0.44 (0.43–0.44)	N/E	0.310
Plasma free-Hb (mg/dL)	28.1 (23.1– 42.5)**	0.42 (0–11.8)	N/E	<0.01
Free-Hb-MECH (mg/dL)	0.24 (0.05–0.58)**	0.1 (0–0.11)	N/E	<0.01

Values are presented as Median (25–75% IQR). Bold: defines statistical significance *p < 0.05, **p < 0.01, patient’s vs. controls. Bil, Bilirubin; plasma free-Hb, plasma free hemoglobin; Free-Hb-MECH, Free Hb after cells’ exposure to mechanical stress; Hb, hemoglobin; Hct, hematocrit; hs CRP, high sensitive C-reactive protein; LDH, lactate dehydrogenase; MCF: mean cell fragility index; MCH, mean corpuscular hemoglobin; MCHC, mean corpuscular hemoglobin concentration; MCV, mean corpuscular volume; MPV, mean platelet volume; PLT, platelets; PDW, platelet distribution width; RBC, red blood cells; RDW, RBC distribution width; WBC, white blood cells. N/E, not existing. NR, normal range; IQR, interquartile range; SD, standard deviation.

**TABLE 2:** Red blood cell characteristics in SARS-CoV-2 patients and in healthy subjects.

	Patients (*N* = 36)	Healthy controls (*N* = 18)	
RBC characteristics	Median (25–75% IQR)	Median (25–75% IQR)	*P*
iCa^2+^ (MFI)	1141 (935–1364)**	743 (606–927)	**<0.01**
RBC-G6PDH (U/g Hb)	23.4 (18.2–32.4)*	16.9 (13.3–26.6)	**0.03**
iROS (MFI)	439 (400– 534)**	544 (482–590)	**<0.01**
RBC caspase-3 (%)†	0.48 (0.24–0.54) (*N* = 13)	0.34 (0.25–0.48) (*N* = 11)	0.38
IgG^+^ RBCs (%)†	0.6 (0.49–0.77) (*N* = 14)	0.55 (0.45–0.65) (*N* = 11)	0.21
PS^+^ RBCs (%)	0.73 (0.55–0.84)**	0.53 (0.44– 0.63)	**<0.01**

Values are presented as Median (25–75% IQR). Bold: pathological values (out of normal range). *p < 0.05, **p < 0.01, patients vs. controls. iCa^2+^, RBCs intracellular calcium; iROS, RBCs intracellular ROS levels; MFI, mean fluorescence index. (†) measured in a subset of patients.

**FIGURE 1:**
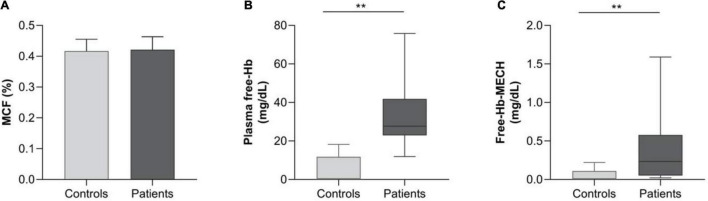
Presenting hemolysis markers in patients (PTs) compared to the control group (CTLs). **(A)** The bar graph illustrates the percentage of mean cell fragility (MCF) in the two groups (patients vs. controls). Box and whisker plot [using the Tukey method] illustrating: **(B)** plasma free Hemoglobin (Hb) in mg/dL in patients vs. controls and **(C)** free Hb after cells’ exposure to mechanical stress (Free-Hb-MECH). **p < 0.01.

Regarding the examination of hemolysis markers, the most striking finding was the markedly elevated plasma free-Hb levels, 28.1g/dL (23.1– 42.5) vs. 0.42 g/dL (0–11.8) (median IQR) in the patients vs. control group, although not being accompanied by a difference in Hb or Indirect Bilirubin. It was, however accompanied by an increase in LDH and Free-Hb-MECH compared with healthy controls (Table 1 and Figure 1). Representative CT images of patients with 0–25, 25–50, and 50–75% CT chest infiltrations are depicted in Supplementary Figure 1.

Intracellular calcium ions levels (iCa^2+^) were increased in SARS-CoV-2 patients compared to healthy controls in our study. In contrast to the equal percentages of IgG^+^ and caspase-3 positive RBCs, indicating the presence of senescent neoantigen and an eryptosis marker respectively, the percentage of RBCs exposing PS was significantly increased in patients compared to the control group. Also, a significant and remarkable increase in the levels of G6PDH activity in patients’ RBCs was also observed. Furthermore, the iROS were significantly decreased in the COVID-19 patients (Table 2).

**FIGURE 2:**
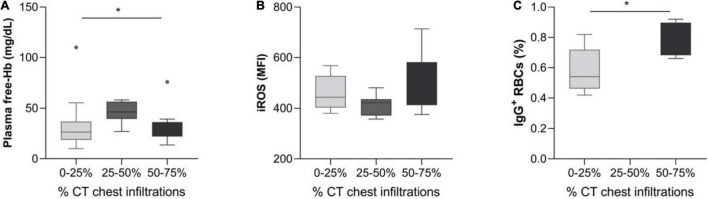
Hemolysis and erythrocyte characteristics in SARS-CoV-2 patients, grouped by chest CT infiltrates. Box and whisker plot [using the Tukey method] illustrating: **(A)** Plasma free-Hb, **(B)** iROS, and **(C)** IgG^+^ RBCs. *p < 0.05, **p < 0.01.

Plasma Free-Hb = 26.5g/dL (18.8–36.8) was noted for patients with 0–25% infiltrations on chest CT upon admission, whereas 46.4 g/dL (39.5–56.4) and 25.3 g/dL (22.1–36.3) values were observed for CT infiltrates 25–50 and 50–75%, respectively (*p* = 0.033). Red blood cells iROS levels showed a tendency of association with chest CT infiltrates [443 (403–527) vs. 421 (371–425) vs. 535 (412–582) for infiltrates 0–25, 25–50, and 50–75%, respectively (*p* = 0.056)] (Figures 2A,B).

**FIGURE 3:**
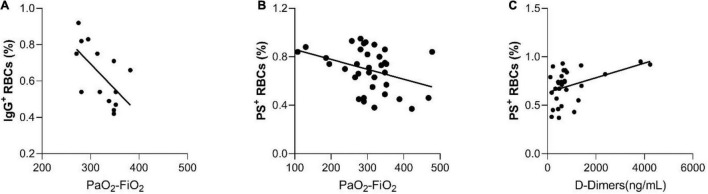
The significant correlations between hemolysis and RBC markers with disease severity, as it is defined by PaO2/FiO 2 ratio. **(A)** Correlation of IgG^+^ RBCs with the PO_2_/FiO_2_ ratio, **(B)** Correlation of PS^+^ RBCs with the PO_2_/FiO_2_ ratio, and **(C)** Correlation between D-Dimers and PS^+^ RBCs.

A significant correlation of IgG^+^ RBCs and the PO_2_/FiO_2_ ratio was also observed (Figure 3A, spearman’s Rho = −0.62, *p* = 0.018). The percentage of IgG^+^ RBCs was markedly elevated in patients with 50–75% chest CT infiltrations compared with those with 0–25% [0.79 (0.68–0.90) vs. 0.54 (0.46–0.72) respectively], *p* = 0.028. Due to the small number of RBC-IgGs measurements (*N* = 14), there were no patients with measured RBC-IgGs and 25–50% chest CT infiltrations (Figure 2C). Moreover, patients’ D-Dimers were found to be associated with PS^+^ RBCs (Figure 3C, *p* = 0.01, Spearman’s Rho = 0.46, *N* = 32) but not with chest CT infiltrations (*p* = 0.17). In addition, PS^+^ RBCs was marginally associated with PaO_2_/FiO_2_ ratio (Spearman’s R = −0.32, *p* = 0.06) (Figure 3B). All associations of RBC markers with disease severity (defined by PaO_2_/FiO_2_ ratio) are depicted in Figure 3.

## Discussion

This pilot study provides novel findings suggesting a link between SARS-CoV-2 and RBC physiology and associates RBC markers with disease severity.

In the present study, SARS-CoV-2 patients have no differences in the majority of hematological and biochemical parameters studied compared to controls (Table 1), however they exhibit raised LDH levels. The increased LDH levels in this group of patients is proposed to be a sign of disease progression and can probably be used for the prediction of worst outcome (Henry et al., 2020; Szarpak et al., 2021). Lymphopenia and increased ferritin and D-dimers levels have also been proposed as predictive markers of disease severity and mortality (Gomez-Pastora et al., 2020; Tavakolpour et al., 2020; Lee et al., 2021; Poudel et al., 2021). The SARS-CoV-2 group of patients in our study actually show all the above characteristics. However, a correlation between these markers and disease severity was not observed, probably as a result of the small number of patients. Increased fibrinogen levels probably reflect the inflammatory state of the patients, as it is indicated by the positive correlation between fibrinogen with CRP in our study (data not shown). The presence of excess iron, as demonstrated by increased serum ferritin in our study might further promote thrombosis in COVID-19 patients, by increasing the clot’s resistance to fibrinolysis (Kell and Pretorius, 2015).

Our results demonstrated that SARS-CoV-2 patients’ plasma free Hb levels were markedly elevated in the patients’ group. Plasma free Hb levels, a marker for intravascular hemolysis is not routinely used in clinical practice, a fact that could explain why RBC hemolysis has not been described in SARS-CoV-2 patients. However, increased plasma free-Hb levels in SARS-CoV-2 have been reported before (Lancman et al., 2021). There is an argument of whether increased plasma free-Hb levels could be attributed to suboptimal venipuncture. Increased plasma free-Hb levels can occasionally be observed in hospitalized patients, as values between 5 and 15 mg/dL, are considered likely to result from suboptimal venipuncture (Lancman et al., 2021). However, in our study, only 2 out of 36 patients had plasma free-Hb lower than 15 (between 10 and 15), leading to the assumption that this marker is probably the result of the effect of the virus on the RBC and not of the suboptimal venipuncture.

Our research suggests that the virus induces RBC oxidative stress, increases intracellular Ca^2+^ and cell’s fragility to mechanical stress. The exact sequence of events cannot be elucidated. However, increased RBC iROS secondary to sepsis have been known to result in decreased RBC deformability, which consists of cell shrinkage, membrane blebbing and cell membrane scrambling, a process referred to as eryptosis (Pretorius, 2018). This may be translated into increased mechanical fragility and RBC lysis, as observed in our case (Machiedo et al., 1989).

D-Dimers are fibrin degradation fragments and their values are associated with thrombosis and fibrinolysis. In our study, there was an association between PS^+^RBCs and D-Dimers (Figure 3B) in the patients’ population suggesting that the RBCs may be involved in COVID-19-associated thrombotic mechanisms. The increase of the intracellular Ca^2+^ levels is known to cause the translocation of membrane phosphatidylserine to the outer space of the RBC membrane (Seigneuret and Devaux, 1984; Bucki et al., 1998), an effect known to stimulate thrombosis through the formation of microparticles and the prothrombinase complex assembly (Kamp et al., 2001). Another possible thrombotic mechanism driven by the RBCs might be the consumption of endothelium-derived nitric oxide by the plasma free-Hb, as previously described, leading to the impairment of endothelium-dependent vasolidation (Loscalzo, 2001).

Surprisingly, red blood cells intracellular ROS levels were significantly lower in the patient group compared to healthy controls. This finding possibly correlates with the increased G6PDH activity levels in this group, suggesting a compensatory mechanism against the virus. This compensatory mechanism seems to be attenuated as chess CT infiltration increases, as shown in Figure 2.

Another intriguing finding is the correlation of RBC-IgGs and the PO_2_/FiO_2_ ratio (Figure 3A), indicating that the increase of IgGs on the RBC surface, is associated with reduced patients’ respiratory capacity. The underlying mechanisms could be either that an aged RBC cannot transport O_2_ very effectively, or that the lack of oxygen supply may cause oxidative RBC damage that leads to RBC senescence. In support of the second mechanism is the increase in percentage of RBC^+^ IgGs in patients with 50–75% compared to 0–25% chest CT infiltrates. In any case, it has been suggested by Berzuini et al. (2020), that the RBC surface of COVID-19 patients is modified due to hyperinflammation, thereby enhancing the deposition of IgG. Our finding of increased RBC-IgGs with disease severity are in support of this assumption.

The association between plasma free Hb and chest CT infiltrations (Figure 2A) is not quite clear, as it appears that plasma free-Hb in patients with 50–75% infiltrates return to levels observed for patients with 0–25% infiltrates. Free hemoglobin in the alveolar space is known to increase iROS probably leading to acute respiratory distress syndrome (ARDS), while its attachment to the alveolar epithelium induces inflammation (Janz and Ware, 2015). Increased mechanical fragility as in our patients’ group might cause RBC lysis as the cells travel along the lung capillaries and subsequent alveolar damage. However, further studies are needed to prove this assumption.

## Limitations

This study has two limitations. The small number of participants and the random timepoints of sampling. Therefore, disease severity as estimated at baseline was associated with RBC markers retrieved from various time points during hospitalization. Even so, associations were found that need further exploitation.

## Conclusion

This pilot study suggests that SARS-CoV-2 infection has an effect on red blood cells and there seems to be an association between RBC markers and disease severity in these patients. These effects need to be further validated in future studies and the causative mechanisms underlying the associations need to be elucidated.

## Data Availability Statement

The raw data supporting the conclusions of this article will be made available by the authors, without undue reservation.

## Ethics Statement

This study was conducted according to the guidelines of the Declaration of Helsinki, and approved by the Institutional Review Board (or Ethics Committee) of National and Kapodistrian University of Athens (decision number: 145/8-3-2021 on 08-04-2021). The patients/participants provided their written informed consent to participate in this study.

## Author Contributions

AK, VP, and SV: methodology, supervision, and project administration. AB, AK, HG, and SF: formal analysis. AB, AK, VP, and SV: investigation. AK, VP, SV, and KS: resources. AK and AB: writing–original draft preparation. TT, LL, KM, DG, APa, and SP: data collection. AB, APo, and EP: statistics. All authors have read, review, editing and agreed to the published version of the manuscript.

## Conflict of Interest

The authors declare that the research was conducted in the absence of any commercial or financial relationships that could be construed as a potential conflict of interest.

## Publisher’s Note

All claims expressed in this article are solely those of the authors and do not necessarily represent those of their affiliated organizations, or those of the publisher, the editors and the reviewers. Any product that may be evaluated in this article, or claim that may be made by its manufacturer, is not guaranteed or endorsed by the publisher.
