# Supplementary material for: Red Blood Cell Abnormalities as the Mirror of SARS-CoV-2 Disease Severity: A Pilot Study
Source: Front Physiol. 2022 Jan 20;12:825055. doi: 10.3389/fphys.2021.825055 (PMC8812589; doi:10.3389/fphys.2021.825055)
Supplement: Supplementary file 1 [file Presentation_1.PPTX]

## Slide 1
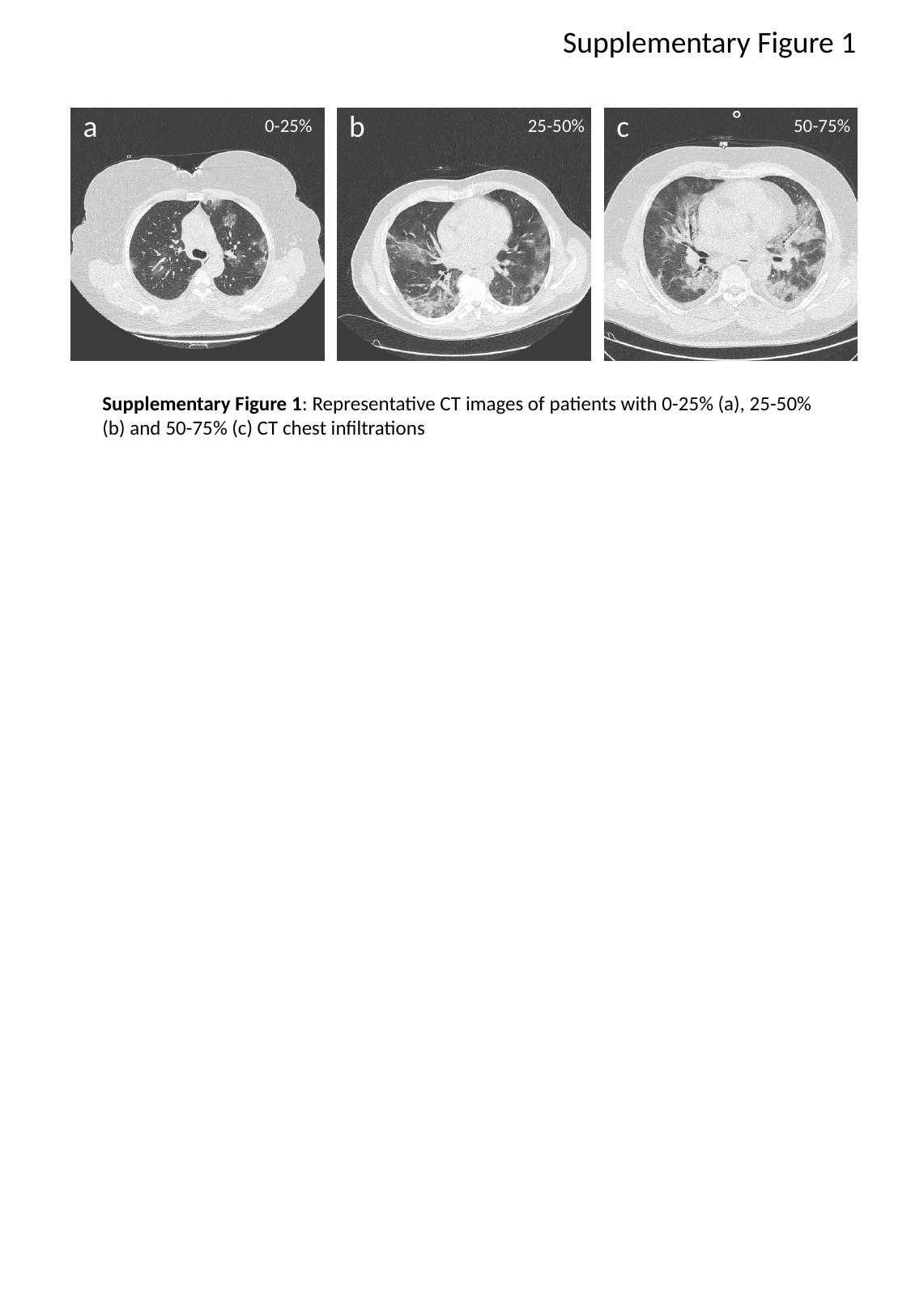

Supplementary Figure 1
a
b
c
0-25%
25-50%
50-75%
Supplementary Figure 1: Representative CT images of patients with 0-25% (a), 25-50% (b) and 50-75% (c) CT chest infiltrations
